# PReS-FINAL-2007: Adolescent clinic for young patients with juvenile idiopathic arthritis

**DOI:** 10.1186/1546-0096-11-S2-P20

**Published:** 2013-12-05

**Authors:** SK Sørensen

**Affiliations:** 1 Peadiatric Department A3, Aarhus University Hospital, Aarhus N, Denmark

## Introduction

Parents are very involved in the treatment and care of their child with juvenile idiopathic arthritis (JIA). As the child grows older, the dynamics in the family should change by the parents moving into the background and the adolescents gradually taking over the responsibility for the treatment and care.

It may be a challenge for the adolescent, the parents and the health care professionals to make the adolescent more involved and to make the parents gradually withdraw.

## Objectives

The purpose is to increase focus on the individual adolescent. Through teaching, the young patient will gradually be prepared to take responsibility and gain knowledge of the disease, treatment and its influence on their future.

Also the parents will slowly hand over the responsibility to the acolecent.

## Methods

An adolescent clinic for patients aged 12-15 years will be established.

## Results

The adolescents are expected to gain a greater understanding of their disease. The adolescents will gradually be introduced to take over the responsibility for their treatment through learning about the disease, the treatment, the impact of the disease on their everyday life and the future. Concurrently, the parents will be supported in handing over the responsibility to their adolescent.

## Conclusion

The transfer from pediatric to adult department will occur smoothly and the experience of transition will improve. The young patients will experience a more youth-oriented approach to their disease and treatment. Which will benefit the transition from childhood to adulthood with JIA.

## Disclosure of interest

None declared.

